# Leisure-time and commuting physical activity and subsequent sickness absence among young and early midlife employees: a register-linked follow-up study 2017–2020

**DOI:** 10.1007/s00420-026-02234-2

**Published:** 2026-09-01

**Authors:** Ville Päivärinne, Pi Fagerlund, Jouni Lahti, Ossi Rahkonen, Tea Lallukka

**Affiliations:** 1 Department of Public Health, University of Helsinki, Helsinki, Finland; 2 Finnish Institute for Health and Welfare, Helsinki, Finland

**Keywords:** Leisure-time physical activity, Active commuting, Gender, Sickness absence

## Abstract

**Purpose:**

We examined leisure-time physical activity (LTPA) and active commuting (AC) and their associations with subsequent sickness absence (SA) among young and early midlife employees.

**Methods:**

The 2017 Helsinki Health Study survey among employees of the City of Helsinki aged 19–39 was prospectively linked to register-based data on total, short- and long-term SA spells (2017–2020). LTPA (n=3618) and AC (n=3513) were assessed as weekly metabolic equivalent (MET) hours in 2017. LTPA was categorized into five groups based on intensity and weekly volume (inactivity, moderate, moderate-to-vigorous, vigorous and high vigorous) and AC into four groups (inactive, low moderate, moderate-to-vigorous, vigorous). Analyses were stratified by sex and adjusted for sociodemographic and lifestyle factors using negative binomial regression to estimate incidence rate ratios (IRRs) and their 95% confidence intervals (CIs).

**Results:**

Women had more SA than men (135 vs 97 days per 10 person-years;* p*<0.001). Women with moderate-to-vigorous LTPA had, compared to the inactive, lower rates of total SA (IRR 0.69, 95% CI 0.60–0.80) short SA and long SA after full adjustments for covariates. Men with vigorous LTPA remained protective in total SA days (IRR 0.60, 95% CI 0.44–0.81) and short SA spells after adjustment. For AC, protective associations were observed only among women.

**Conclusion:**

LTPA including vigorous-intensity activity was associated with lower SA over the follow-up, although patterns differed by sex and activity level. Associations for AC were weaker and confirmed for women only. Vigorous-intensity physical activity may be beneficial for reducing SA already in early working life.

**Supplementary information:**

The online version contains supplementary material available at https://doi.org/10.1007/s00420-026-02234-2.

## Introduction

Sickness absence (SA) represents a major public health, economical and societal challenge in Western countries. It imposes substantial costs on employees, workplaces, and society as a whole (Lallukka et al. 2020a, b; Virtanen et al. 2018). In addition, SA due to mental health problems have increased markedly, especially among women (Blomgren and Perhoniemi 2022). Overall, higher levels of SA have been reported in female-dominated workplaces, suggesting that gender-related factors play and important role in shaping SA patterns(Haukenes and Hammarström 2024). Perceived work ability has declined among younger age groups in Finland during the last two decades and consequently work ability is projected to decline among middle-aged and older age groups in the becoming decades (Lahti et al. 2025).Therefore, effective strategies to counteract declining work ability at the population level are urgently needed.

Recent evidence indicates that approximately 31% of the world’s population does not meet the World Health Organization’s (WHO) minimum recommendations for physical activity (2,5 h of moderate intensity physical activity per week), contributing to considerable economic burdens on healthcare and social welfare systems worldwide (Bull et al. 2020). Physical inactivity has been identified as a risk factor for numerous chronic diseases and is one of the leading contributors to global mortality (Ding et al. 2020). Regular leisure-time physical activity (LTPA) has been associated with 1–2 additional disease-free years, with the largest gains observed among individuals at elevated health risk, particularly those with lower socioeconomic position (Nyberg et al., 2025). A recent meta-analysis estimated that increasing moderate-to-vigorous LTPA by as little as 5 min per day could reduce population mortality by up to 10% (Ekelund et al. 2026). Active commuting (AC) has been associated with lower all-cause and cardiovascular mortality in meta-analyses, reduced cardiovascular disease risk and improved cardiorespiratory fitness and work ability (Dutheil et al. 2020; Hamer and Chida 2008; Kalliolahti et al. 2024).

Among Finnish employees, work ability has been observed to decline already between the ages of 31 and 46, emphasizing the importance of promoting work ability and identifying risk factors for its deterioration at an early stage (Punakallio et al. 2019). Evidence remains particularly limited regarding studies examining both LTPA and AC and risk of subsequent SA spells of different length. Addressing these gaps is important for informing early preventive strategies in the working-age population. Previous research has shown that LTPA is associated with a reduced risk of SA (Ketels et al. 2023; Lahti et al. 2012; López-Bueno et al. 2020; Proper et al. 2006) whereas there is less evidence regarding AC on relation to SA (Kalliolahti et al., 2025; Mytton et al. 2016). While physical activity is widely acknowledged as a determinant of health and work ability, little is known about how different domains, volumes, and intensities of activity translate into SA outcomes.

Health behaviors and health outcomes are unequally distributed across population groups. Individuals with lower socioeconomic position tend to report poorer perceived health and lower work ability compared to those with medium or higher socioeconomic position (Laaksonen et al. 2011). High mental and physical demands have been associated with a higher risk of SA (Andersen et al. 2016). In addition, factors that help maintain work ability include healthy lifestyle behaviors, and normal body weight (De Bortoli et al. 2021). The aim of this study was to examine how different volumes and intensities of LTPA and AC relate to subsequent SA (total SA days, and days of short and long SA spells, respectively), and whether these associations differ between women and men, controlling for key covariates such as age, occupational class, mental or physical strenuousness of work, smoking and body mass index (BMI).

## Methods

### Study population

**Fig. 1:**
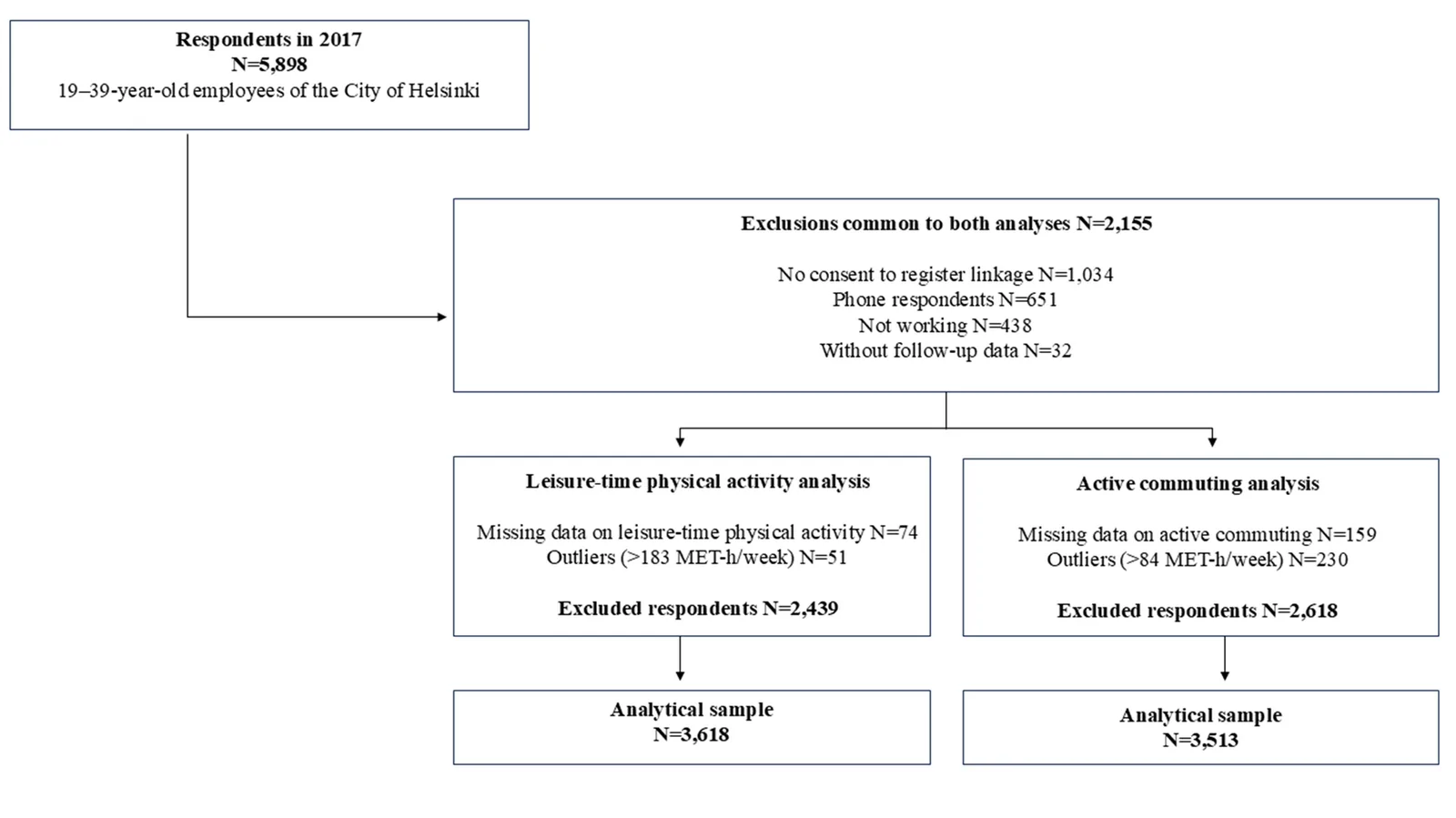
Flow chart of the Helsinki health study population 2017–2020

This was a longitudinal prospective cohort study based on data collected between 2017 and 2020. The analyses utilized questionnaire data from the Helsinki Health Study (HHS) combined with prospectively linked register-based SA information from the City of Helsinki, Finland. The survey was conducted in autumn 2017 (Phase 1) among employees of the City of Helsinki aged 19–39 years who were eligible at the time of sampling, defined as having an employment contract of at least 50% for a minimum of four months prior to sampling. (*n* = 11,459) (Fig. 1). Data were gathered, including online questionnaires (58%), postal surveys (29%) and telephone interviews (13%) (Lallukka et al. 2020a, b). Participants with a registered email address were invited to complete the survey online, while paper questionnaires were sent to those without email access. All individuals who did not respond after the initial reminders were mailed a paper questionnaire containing login information in the cover letter, allowing them to select their preferred mode of response (online or via mail); postage costs were prepaid. The overall response rate was 51% (*n* = 5898), and women comprised 79% of the respondents, closely reflecting the sex distribution of the target population (and the Finnish municipal workforce) (Lallukka et al. 2020a, b). Consent to register linkage (81%, *n* = 4864) was required for inclusion in the study population. Participants who were interviewed by telephone (*n* = 651) were excluded, because the interview did not comprise the variable of interest of this study. Additionally, those who were not working full- or part-time at baseline (*n* = 438), without follow-up data (*n* = 32) and with missing LTPA (*n* = 74) or AC (*n* = 159) were excluded. The cut-off for removing the outliers was determined using the z-score method: a z-score of 3 was considered the threshold(Anusha et al. 2019). We chose the analyses to a common subset of respondents with complete data for both exposures, in order to maximize statistical power for each exposure-specific analysis. Therefore, as LTPA being the exposure, the final dataset consisted of 2850 women and 768 men. As for AC being the exposure, the final dataset consisted of 2773 women and 740 men.

## Exposures

### Leisure-time physical activity and active commuting

Baseline (2017) weekly LTPA and AC were assessed separately with four items capturing activities at four intensity levels. Participants reported the number of hours per week spent in each intensity category. The items corresponded to low-intensity activity (walking), moderate intensity (brisk walking or equivalent), vigorous intensity (such as jogging) and high vigorous intensity (running). Each intensity level was assigned a metabolic equivalent (MET) value (Kujala et al. 1998), which was used to calculate weekly MET-hours for the four intensity categories. The questionnaire items used to assess LTPA and AC are presented in Supplementary File 1. Following this, LTPA was categorized into five groups and AC into four groups based on distributional cut-offs with additional intensity conditions. LTPA groups were defined as: inactivity (< 14.0 MET-h/week without vigorous activity), moderate (14.0–39.9 MET-h/week without vigorous), moderate-to-vigorous (14.0–39.9 MET-h/week with vigorous), vigorous (40.0–69.9 MET-h/week with vigorous) and high vigorous (≥ 70.0 MET-h/week with vigorous). AC groups were defined as: inactivity (< 3.0 MET-h/week without vigorous), moderate (3.0–8.9 MET-h/week without vigorous), moderate-to-vigorous (9.0–19.9 MET-h/week with vigorous) and vigorous (≥ 20.0 MET-h/week with vigorous).

### Outcome measure: sickness absence

Information on SA was obtained from the City of Helsinki personnel register for employees who consented to record linkage (82%). SA data were obtained from the employer (the City of Helsinki) personnel register for the employees who consented to record linkage, and that the register data were linked to questionnaire survey responses using unique personal identifiers assigned to each Finnish citizen at birth. Follow-up began on the day after the questionnaire was returned and continued through March 2020 (coinciding with the onset of the COVID-19 pandemic in Finland) or until the end of the employment contract, whichever occurred first (mean follow-up was 2.2 years). We analyzed total SA days and, in addition, differentiated short SA spells (1–7 days) from long SA spells (≥ 8 days), as one week represents the maximum duration for self-certified absence spells recorded in the employer register, which could be approved by supervisors without a medical certificate. During the follow-up, SA of eight days or longer required a physician-approved medical certificate, and the same SA policy applied to all City of Helsinki employees. Additionally, we analyzed SA of all lengths combined.

### Covariates

Covariates were derived from the survey. Three age groups were used: 19–29, 30–34, and 35–39 years at Phase 1. We classified sex as women and men. Occupational class was classified into three categories: manual or routine non-manual workers’ (e.g., care workers and underground train drivers), ‘semi-professional’ (e.g., nurses and technicians) and ‘professional’ (e.g., teachers, doctors, and managers). The classification reflects occupational hierarchy and job characteristics rather than professional status. Physical and mental strenuousness of work were assessed using single-item questions asking participants to rate how physically or mentally strenuous their job was. Response options were ”Very light”, “Rather light”, “Rather strenuous”, and “Very strenuous”. Responses were categorized into three levels for analyses: low (very/rather light), moderate (rather strenuous), and high (very strenuous). BMI was calculated from weight and height (kg/m^2^), and participants were categorized into four groups those with underweight (< 1%) and healthy weight (BMI ≤ 25.0 kg/m^2^), those with overweight (25.0–29.9 kg/m^2^), obesity (BMI < 30 kg/m^2^), and a separate category for missing BMI. Smoking was divided into smokers (daily and occasional) and non-smokers (non-smokers and ex-smokers).

### Statistical methods

Cross-tabulation with chi-squared tests were used to describe and compare the Phase 1 characteristics among women and men (Table 1). We used a negative binomial model to calculate incidence rate ratios (IRR) and their 95% confidence intervals (CI) to examine the association with SA to different volumes and intensities of LTPA and AC. Predicted marginal means per 10 person-years from the crude negative binomial regression models represent expected values standardized for follow-up time (Table 2 & Supplementary Tables 1–2). The ‘exposure’ groups were formed based on LTPA or AC, separately. Inactive participants in LTPA and AC were used as reference group. All models in the main analysis were adjusted for age with an offset for follow-up time at work. Additional adjustments for individual covariates were performed (Supplementary Tables 3, 4, 5, 6). To avoid loss of individuals due to missing BMI and to retain comparability across models, individuals with missing BMI were included as a separate category in the adjusted analyses.

Neither of the interaction terms between LTPA or AC and sex reached statistical significance when formally tested (LTPA = 0.08; AC = 0.30). However, analyses were stratified by sex instead of pooled analyses to provide a clearer understanding of how physical activity may relate to SA separately in women and men.

**Fig. 2:**
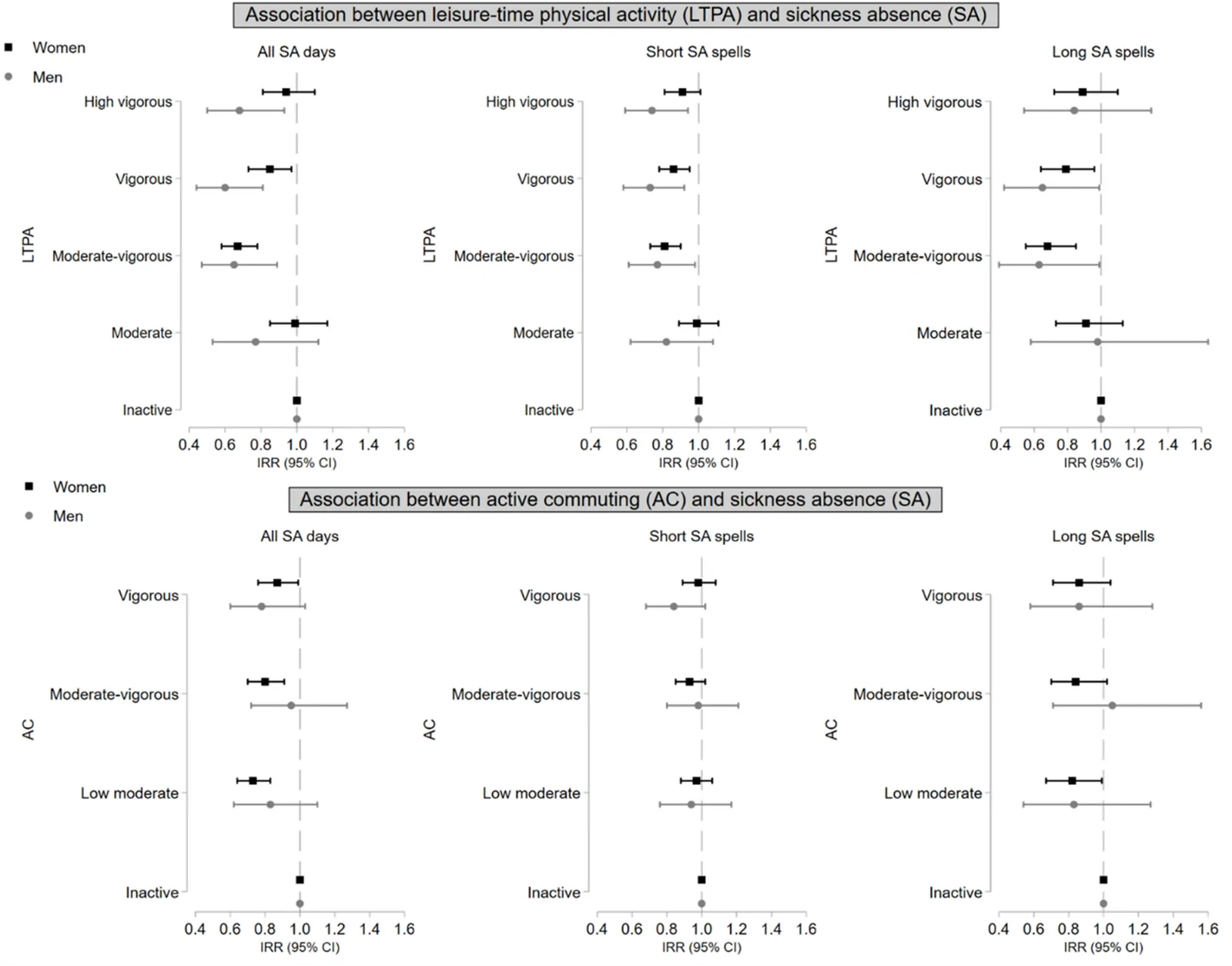
Incidence rate ratios (IRR) with 95% confidence intervals (CIs) from negative binomial regression models for the associations of leisure-time physical activity (LTPA) levels (upper panels) and active commuting (AC) levels (lower panels) with sickness absence (SA) outcomes: total SA days (left column), short SA spells (middle column), and long SA spells (right column) between sex. Inactivity was used as the reference category. Estimates are derived from negative binomial regression models, adjusted for age and follow-up time (offset)

We first present sex-stratified associations between physical activity and SA as the primary analyses. Interaction models were fitted next to formally assess effect modification by sex and to illustrate predicted marginal means. Predicted marginal means presented in Fig. 2 were estimated from models including interactions between sex and LTPA or AC to illustrate potential sex-specific patterns in SA per 10 person-years. Consequently, predicted means were higher than the descriptive averages, due to skewed distributions of SA days. As an additional analysis, potential interaction between LTPA and AC was examined with data available for both exposures (*n* = 3488) by including a multiplicative interaction term between the categorial LTPA and AC variables in an age- and sex-adjusted negative binomial regression model.

The STROBE statement checklist is presented in Supplementary File 2.

Stata 19.5 (StataCorp LP; College Station, Texas, USA) statistical package was used for all analyses.

## Results

Women comprised approximately four-fifths of the study population (Table 1). Compared with men, women were younger, more often employed in semi-professional occupations, and reported lower levels of both mental and physical strenuousness of work (*p* < 0.001). Men were more frequently employed in manual or routine non-manual occupations and had higher BMI, with overweight being more common among men than women (*p* < 0.001) Smoking prevalence did not differ between women and men. Regarding physical activity, men more often reported high vigorous LTPA whereas women were more frequently inactive (*p* < 0.001) No statistically significant sex differences were observed in AC categories (*p* = 0.062).

Women reported higher levels of SA than men (Table 2), including total SA days as well as both short and long SA spells (*p* ≤ 0.001). More precisely, women had more SA days per 10 person-years than men in both LTPA (135.1 days [128.7–141.5] vs. 95.1 days [86.5–103.8]) and AC (134.8 days [128.3–141.3] vs. 95.8 [86.9–104.7]) datasets.

In the main analysis, among women (Fig. 2), moderate-to-vigorous LTPA was associated with significantly fewer SA days compared with inactivity (IRR 0.67, 95% CI 0.58–0.78) after adjusting for age. The association was weaker at vigorous activity levels (IRR 0.85, 95% CI 0.73–0.97) and was no longer evident at high vigorous LTPA, suggesting a non-linear association. Among men, moderate-to-vigorous and higher levels of LTPA were consistently associated with fewer SA days compared with inactivity (moderate-to-vigorous LTPA : IRR 0.65, 95% CI 0.47–0.89; vigorous LTPA: IRR 0.60, 95% CI 0.44–0.81; high vigorous LTPA: IRR 0.68, 95% CI 0.50–0.93), with no clear attenuation of the association at higher activity levels. Additionally, among women, the association between moderate-to-vigorous LTPA and SA, and among men, the association for vigorous LTPA, persisted after additional adjustment for individual covariates (Supplementary Tables 3 and 4). The overall pattern of associations was similar for short and long SA spells. However, the association between high vigorous LTPA and long SA spells did not persist for among men after adjustment for occupational class and BMI.

Among women, AC was associated with total SA, when compared with inactivity and adjusted for age (low moderate AC: IRR 0.73, 95% CI 0.63–0.83; moderate-vigorous AC IRR 0.80, 95% CI 0.70–0.91; vigorous AC IRR 0.87, 95% CI 0.76–0.99). The association between low moderate and moderate-to-vigorous AC persisted after additional adjustment for individual covariates (Supplementary Tables 4 and 5). No statistically confirmed associations were found among men (Supplementary Table 6).

**Fig. 3:**
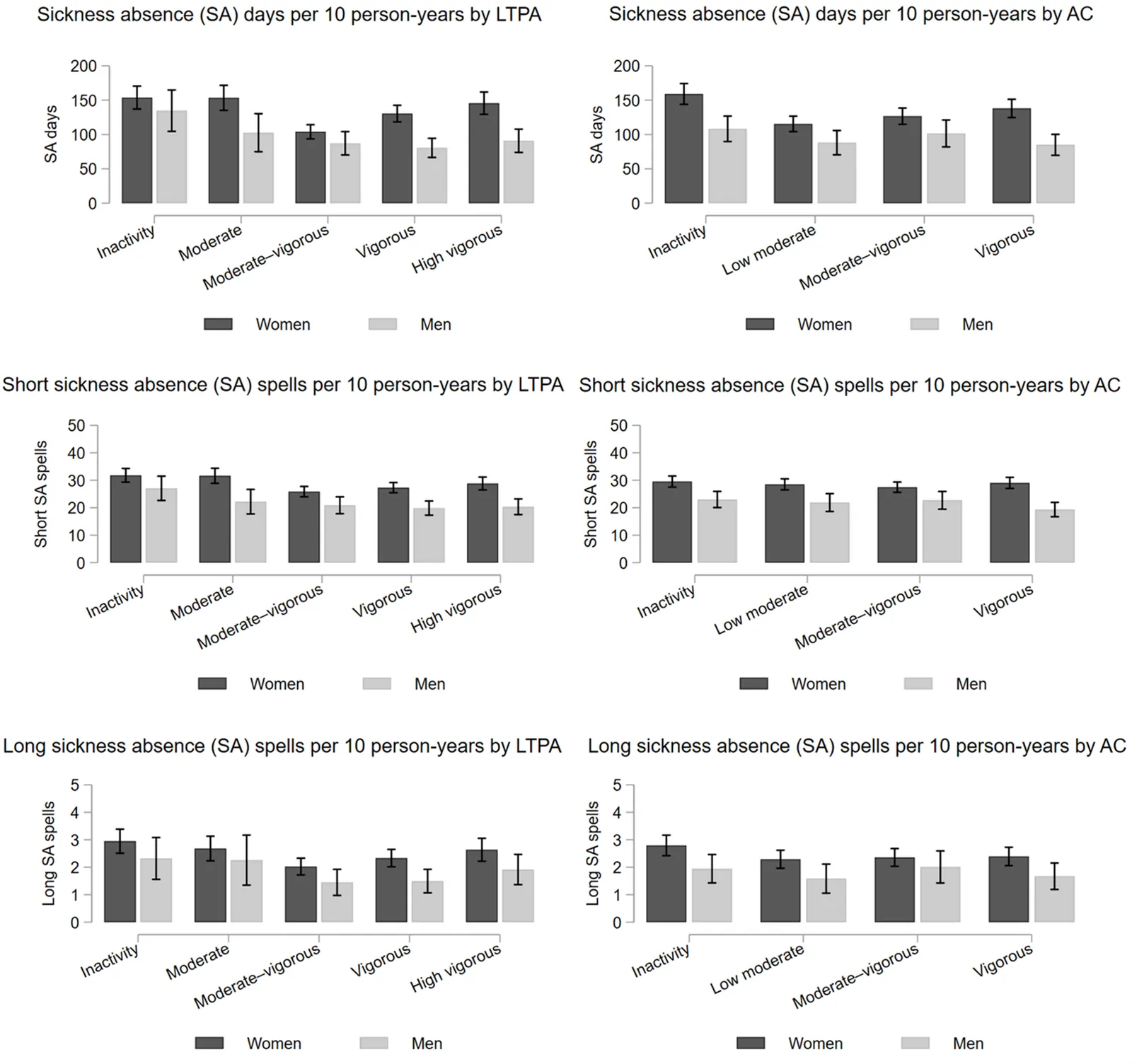
Model-predicted mean number of sickness absence (SA) days, short SA spells and long SA spells per 10 person-years by leisure-time physical activity (LTPA) and active commuting (AC) by sex. Estimates are derived from negative binomial regression models including sex and LTPA or AC interactions, adjusted for age and follow-up time (offset). Error bars indicate 95% confidence intervals

Among women, when adjusted for age (Fig. 3), moderate-to-vigorous LTPA activity was associated with fewer SA days per 10 person-years compared with inactivity (moderate-to-vigorous: 104.0 days [95% CI 93.7–114.3] vs. inactivity: 145.6 days [95% CI 129.3–161.8]). Estimates at higher activity levels were closer to those of the inactive group, indicating attenuation of the association at higher LTPA levels (Supplementary Table 7). As for AC, low moderate (115.5 days [95% CI 104.3–126.7) and moderate-to-vigorous (126.7 days [114.8–138.5]) AC were associated with fewer SA days per 10 person-years compared with inactivity (159.0 days [95% CI 143.9–174.2]) (Supplementary Table 8).

Among men (Fig. 3), moderate-to-vigorous and higher levels of LTPA were consistently associated with fewer SA days compared with inactivity (moderate-to-vigorous: 87.2 days [70.2–104.2]; vigorous: 80.5 days [95% CI 66.6–94.4] vs. inactivity: 145.6 days [95% CI 129.3–161.8]), with no clear attenuation of the association at higher activity levels (Supplementary Table 7). No significant associations emerged among men in AC. In an additional analysis (data not shown), there was evidence of an interaction between LTPA and AC in relation to SA (P for interaction = 0.007).

**Table 1:** Descriptive statistics by sex, the Helsinki Health Study, 2017 between leisure-time physical activity (*n* = 3618) and active commuting (*n* = 3513).

	Leisure-time physical activity			Active commuting		
	Women (*n*, %)	Men (*n*, %)	*p*	Women (*n*, %)	Men (*n*, %)	*p*
	2850 (79)	768 (21)		2773 (79)	740 (21)	
*Age*			< 0.001			< 0.001
19–29	966 (34)	195 (25)		949 (34)	193 (26)	
30–34	941 (33)	251 (33)		919 (33)	246 (33)	
35–39	943 (33)	322 (42)		905 (33)	301 (41)	
*Occupational class*			< 0.001			< 0.001
Professional	817 (29)	231 (30)		783 (28)	284 (30)	
Semi-professional	1223 (43)	243 (32)		1201 (43)	232 (31)	
Manual/routine non- manual	810 (28)	294 (38)		789 (29)	224 (39)	
*Mental strenuousness of work*			< 0.001			< 0.001
Low	527 (19)	225 (29)		509 (18)	212 (29)	
Moderate	1829 (64)	409 (53)		1773 (64)	401 (54)	
High	494 (17)	134 (18)		491 (18)	127 (17)	
*Physical strenuousness of work*			< 0.001			< 0.001
Low	628 (22)	200 (26)		615 (22)	192 (26)	
Moderate	1266 (44)	374 (49)		1217 (44)	361 (49)	
High	956 (34)	194 (25)		941 (34)	187 (25)	
*Body mass index*			< 0.001			< 0.001
<25.0 kg/m^2^	1753 (61)	354 (46)		1706 (61)	345 (47)	
25.0–29.9 kg/m^2^	646 (23)	292 (38)		634 (23)	279 (38)	
≥30.0 kg/m^2^	418 (15)	119 (16)		403 (15)	113 (15)	
Missing	33 (1)	3 (0.4)		30 (1)	3 (0.4)	
*Smoking*			0.38			0.34
No	2163 (76)	571 (74)		2104 (76)	549 (74)	
Yes	687 (24)	197 (26)		669 (24)	191 (26)	
*Leisure-time physical activity*			< 0.001			
High vigorous	506 (18)	182 (24)		–	–	
Vigorous	734 (26)	209 (27)				
Moderate-vigorous	636 (22)	165 (21)		–	–	
Moderate	443 (15)	88 (12)		–	–	
Inactive	531 (19)	124 (16)		–	–	
*Active commuting*						0.062
Vigorous	–	–		686 (25)	196 (26)	
Moderate-vigorous	–	–		721 (26)	169 (23)	
Low moderate	–	–		666 (24)	161 (22)	
Inactive	–	–		700 (25)	214 (29)	

*P*-values present differences between women and men

**Table 2:** Subsequent sickness absence (SA) days and spells by sex per 10 person-years, the Helsinki Health Study, 2017–2020 between leisure-time physical activity (LTPA) (*n* = 3618) and active commuting (AC) (*n* = 3513).

		SA days	SA spells	
			Shorter	Longer
*LTPA*	N	Mean (95% CI)	Mean (95% CI)	Mean (95% CI)
All participants	3618	126.6 (121.2–131.9)	27.2 (26.3–28.0)	2.3 (2.2–2.5)
Women	2850	135.1 (128.7–141.5)	28.7 (27.7–29.7)	2.5 (2.3–2.7)
Men	768	95.1 (86.5–103.8)	21.6 (20.1–23.1)	1.8 (1.5–2.1)
*AC*				
All participants	3513	126.5 (121.1–131.9)	27.1 (26.3–28.0)	2.3 (2.2–2.5)
Women	2773	134.8 (128.3–141.3)	28.6 (27.6–29.6)	2.5 (2.3–2.6)
Men	740	95.8 (86.9–104.7)	21.7 (20.2–23.2)	1.8 (1.5–2.1)

Values are presented in means with 95% confidence intervals (CIs)

## Discussion

Our study conducted among young and early midlife Finnish employees showed that vigorous LTPA, when compared to physical inactivity, was associated with lower rates of SA days during the follow up between 2017 and 2022. Among women, the association was weaker for higher LTPA volume, whereas in men it remained relatively stable across activity levels. After adjusting for covariates, moderate-to-vigorous LTPA among women and vigorous LTPA among men remained associated with lower rates of total, short and long SA days. AC was also associated with fewer SA days among women compared with the inactive group, and these associations largely persisted after adjusting for covariates, although they attenuated in the most active group. No association could be confirmed among men.

Our findings are generally consistent with previous longitudinal studies showing that higher levels of LTPA are associated with fewer SA days (Holtermann et al. 2012; Laaksonen et al. 2009). Some previous studies also suggest that increasing moderate-to-vigorous or vigorous LTPA contribute to lower SA among older working-age adults (Lahti et al. 2012; López-Bueno et al. 2020), including those in the same age range as in our study (Ketels et al. 2023; Proper et al. 2006).

The MET-based estimates reflect both weekly intensity and duration. The finding that moderate-to-vigorous, but not moderate, LTPA was associated with fewer SA days—despite similar total volumes—suggests that intensity may be an important aspect of the relationship. Vigorous activity induces greater physiological adaptations per unit time and may be more accurately reported than moderate or light activity, reducing exposure misclassification (Quinlan et al. 2021; Saint-Maurice et al. 2018). Furthermore, a recent study has shown that the validity of physical activity questionnaires varies by fitness level, with lower-fit individuals being more prone to over-reporting, and BMI acting as a useful proxy for fitness in epidemiological studies (Meh et al. 2023). This mechanism could partly explain why adjusting for BMI attenuated the associations in our data. In an occupational context, vigorous LTPA may more effectively enhance resilience and work ability, which are critical for preventing SA.

The association between LTPA and SA showed sex-specific differences. Among men, the protective association persisted across higher activity categories, which resembles the plateau pattern reported in large dose–response analyses of LTPA and mortality, where health benefits increased up to three to five times the recommended minimum, after which additional volume conferred only modest further benefit, with no evidence of harm and no indication of additional protective effect (Arem et al. 2015). In contrast, among women the strongest association emerged at moderate-to-vigorous levels and attenuated at the highest category, with estimates returning close to unity. This non-monotonic pattern at very high volumes is compatible with large, pooled dose–response study on non-occupational physical activity and major health outcomes, where relative risk reductions tend to flatten and become statistically imprecise at the highest exposure levels (Garcia et al. 2023). This pattern among women may also relate to evidence that women derive comparable or greater survival benefits from lower LTPA doses than males. In a large US cohort analysis, women achieved maximal mortality risk reduction at ~ 140 min/week of moderate-to-vigorous LTPA, whereas men achieved a similar benefit only at 300 min/week; for both women and men, the benefits plateaued thereafter (Ji et al. 2024). However, recent dose–response meta-analyses similarly suggest broadly comparable shapes of the LTPA–mortality curve in men and women, implying that the attenuation observed among women in our study may not be attributable to biological intensity-response differences alone (Ekelund et al. 2024). It is also possible that higher levels of LTPA could have led to increased injury rates (Gabbett 2016), which may partly explain the weaker association between LTPA and SA among women. In addition, women tend to use health care services more frequently than men (Lehto et al. 2024), which may also contribute to higher detection and reporting of health issues, and thus influence SA patterns in our study. Nevertheless, our findings suggest working-age women may reach optimal LTPA benefits at moderate volumes, particularly when accounting for their higher total physical activity load across household and occupational domains (Ainsworth et al. 1993; Harryson et al. 2016). This interpretation aligns with evidence that high occupational physical activity without sufficient recovery can attenuate the health benefits otherwise observed for LTPA (Holtermann et al. 2012). Yet, biological differences in intensity responses (Quinlan et al. 2021), as well as methodological factors including measurement error, residual confounding, and selection, cannot be ruled out as explanations for the observed attenuation among women.

AC displayed a similar pattern of association with SA, although the associations were generally weaker. Among women, low to moderate levels of active commuting were associated with fewer SA days compared with inactivity, whereas no associations could be confirmed among men. AC is typically performed on most workdays and is therefore integrated into daily routines, which means that even relatively short walking or cycling trips can accumulate to a meaningful weekly dose of light-to-moderate physical activity (Dutheil et al. 2020). Previous studies have shown that AC, particularly cycling, is associated with better health, work ability and reduced SA (Kalliolahti et al. 2024, 2025; Mytton et al. 2016). Moreover, our findings are consistent with evidence suggesting that even lower-intensity forms of AC are associated with reduced health risks. A recent systematic review and meta-analysis concluded that AC is associated with all-cause and cardiovascular mortality, with a dose–response relationship particularly evident for walking, which typically reflects light-to-moderate intensity activity (Dutheil et al. 2020). Another meta-analysis reported that walking and cycling-based active commuting was associated with an overall 11% reduction in cardiovascular risk, with stronger associations among women (Hamer and Chida 2008). Thus, the apparent difference between AC and LTPA may reflect differences in regularity and classification of activity rather than differences in the effects of walking or cycling per se.

### Methodological considerations

Some limitations should be acknowledged. Since the study population, and public sector employees in general, consist predominantly of women, statistical power for male-specific estimates was limited. Findings should be only cautiously generalized to male employees outside the municipal sector. However, there is no particular reason to assume why the associations between physical activity and SA would be notably different or inverse in different sectors in Finland. For short, self-certified SA, there are minor differences between companies what is allowed before requiring a medical certificate. However, it is always required for longer absence, and same rules apply across Finland, since the Social Insurance Institution of Finland covers for the employer the cost of long SA in the same way in entire Finland. Furthermore, self-reported measures are prone to misclassification, and physical activity levels are often overestimated due to recall-related inaccuracies. In addition, responses may be influenced by social desirability, whereby participants report behaviors they perceive as appropriate or health-promoting (Corder et al. 2019). Furthermore, self-reported physical activity may distort dose-response relationships compared to objective measures (Niemelä et al. 2019). Although non-response analysis suggested that the respondents broadly represented the target population(Lallukka et al. 2020a, b) and the response rate was acceptable, non-response and potential healthy worker selection remain limitations. In addition, although LTPA and AC were analyzed separately because they represent different domains of physical activity, it is possible that their combined effects or interactions influence SA. Future studies should examine the joint contribution of different physical activity domains. Our additional analysis indicated an interaction between LTPA and AC in relation to SA. This suggests that the association of one physical activity domain with SA may vary according to the level of activity in the other domain, as the nature and potential relevance of this interaction need further investigation.

A major strength of this study is the use of a large employee cohort including young and midlife workers across a wide range of occupations. Another strength is the use of employer register data on SA and not self-reported, periods and the longitudinal design. The data included all SA periods, also short and long spells. This is important because total SA days and short spells are rarely included in national registers and therefore less studied. An additional strength is that both LTPA and AC were examined within the same cohort using the same outcome and follow-up, and the analyses were conducted separately.

These findings add to the growing body of evidence that, compared with inactivity, both LTPA and AC are associated with SA already in early and mid-adulthood. However, the observed sex differences and attenuation of associations at higher activity levels highlight the need for further research to better understand optimal activity patterns for preventing SA across different population groups.

## Conclusion

Our findings highlight the importance of considering both the volume and intensity of physical activity, as well as differences in work and recovery contexts between women and men, when examining associations with SA. Taken together, the results emphasize the potential of promoting physical activity in everyday life as part of workplace health promotion. Such efforts may be especially relevant for young and mid-career employees, for whom early prevention of SA could yield long-term benefits. Moreover, the findings emphasize the relevance of vigorous LTPA for younger working-age adults. Similar associations between vigorous LTPA and reduced SA have previously been observed among midlife and older employees, suggesting that vigorous activity may play an important role for work ability across different stages of working life.

## Supplementary information

Below is the link to the electronic supplementary material.

Supplementary Material 1

## Data Availability

The Helsinki Health Study survey data (and the register data of the City of Helsinki) cannot be made publicly available due to strict data protection statutes and regulations. The data can only be used for scientific research. More information on the survey data can be requested from the Helsinki Health Study research group (kttl-hhs@helsinki.fi).

## References

[CR1] Ainsworth BE, Richardson M, Jacobs DR, Leon AS (1993) Gender differences in physical activity. Women Sport Phys Activity J 2(1):1–16. 10.1123/wspaj.2.1.1

[CR2] Andersen LL, Fallentin N, Thorsen SV, Holtermann A (2016) Physical workload and risk of long-term sickness absence in the general working population and among blue-collar workers: prospective cohort study with register follow-up. Occup Environ Med 73(4):246–253. 10.1136/oemed-2015-103314 26740688

[CR3] Anusha PV, Anuradha C, Murty C, P. S. R., Kiran CS (2019) Detecting outliers in high dimensional data sets using Z-score methodology. Int J Innovat Technol Exploring Eng 9(1):48–53. 10.35940/ijitee.A3910.119119

[CR4] Arem H, Moore SC, Patel A, Hartge P, Berrington de Gonzalez A, Visvanathan K, Campbell PT, Freedman M, Weiderpass E, Adami HO, Linet MS, Lee I-M, Matthews CE (2015) Leisure time physical activity and mortality: a detailed pooled analysis of the dose-response relationship. JAMA Intern Med 175(6):959–967. 10.1001/jamainternmed.2015.0533 PMC4451435 25844730

[CR5] Blomgren J, Perhoniemi R (2022) Increase in sickness absence due to mental disorders in Finland: trends by gender, age and diagnostic group in 2005–2019. Scand J Public Health 50(3):318–322. 10.1177/1403494821993705 PMC9096587 33615899

[CR6] Bull FC, Al-Ansari SS, Biddle S, Borodulin K, Buman MP, Cardon G, Carty C, Chaput JP, Chastin S, Chou R, Dempsey PC, Dipietro L, Ekelund U, Firth J, Friedenreich CM, Garcia L, Gichu M, Jago R, Katzmarzyk PT, Willumsen JF (2020) World Health Organization 2020 guidelines on physical activity and sedentary behaviour. Br J Sports Med 54(24):1451–1462. 10.1136/bjsports-2020-102955 PMC7719906 33239350

[CR7] Corder K, Winpenny E, Love R, Brown HE, White M, van Sluijs E (2019) Change in physical activity from adolescence to early adulthood: a systematic review and meta-analysis of longitudinal cohort studies. Br J Sports Med 53(8):496–503. 10.1136/bjsports-2016-097330 PMC6250429 28739834

[CR8] De Bortoli MM, Oellingrath IM, Fell AKM, Burdorf A, Robroek SJW (2021) Influence of lifestyle risk factors on work ability and sick leave in a general working population in Norway: a 5-year longitudinal study. BMJ Open 11(2):e045678. 10.1136/bmjopen-2020-045678 PMC7925900 33550269

[CR9] Ding D, Ramirez Varela A, Bauman AE, Ekelund U, Lee IM, Heath G, Katzmarzyk PT, Reis R, Pratt M (2020) Towards better evidence-informed global action: lessons learnt from the Lancet series and recent developments in physical activity and public health. Br J Sports Med 54(8):462–468. 10.1136/bjsports-2019-101001 PMC7146932 31562122

[CR10] Dutheil F, Pélangeon S, Duclos M, Vorilhon P, Mermillod M, Baker JS, Pereira B, Navel V (2020) Protective effect on mortality of active commuting to work: a systematic review and meta-analysis. Sports Med (Auckland N Z) 50(12):2237–2250. 10.1007/s40279-020-01354-0 33034873

[CR11] Ekelund U, Sanchez-Lastra MA, Dalene KE, Tarp J (2024) Dose-response associations, physical activity intensity and mortality risk: a narrative review. J Sport Health Sci 13(1):24–29. 10.1016/j.jshs.2023.09.006 PMC10818107 37734548

[CR12] Ekelund U, Tarp J, Ding D, Sanchez-Lastra MA, Dalene KE, Anderssen SA, Steene-Johannessen J, Hansen BH, Morseth B, Hopstock LA, Sagelv E, Nordström P, Nordström A, Hagströmer M, Dohrn I-M, Diaz KM, Hooker S, Howard VJ, Lee I-M, Fagerland MW (2026) Deaths potentially averted by small changes in physical activity and sedentary time: an individual participant data meta-analysis of prospective cohort studies. Lancet (London England). 10.1016/S0140-6736(25)02219-6 41544645

[CR13] Gabbett TJ (2016) The training-injury prevention paradox: should athletes be training smarter and harder? Br J Sports Med 50(5):273–280. 10.1136/bjsports-2015-095788 PMC4789704 26758673

[CR14] Garcia L, Pearce M, Abbas A, Mok A, Strain T, Ali S, Crippa A, Dempsey PC, Golubic R, Kelly P, Laird Y, McNamara E, Moore S, de Sa TH, Smith AD, Wijndaele K, Woodcock J, Brage S (2023) Non-occupational physical activity and risk of cardiovascular disease, cancer and mortality outcomes: a dose-response meta-analysis of large prospective studies. Br J Sports Med 57(15):979–989. 10.1136/bjsports-2022-105669 PMC10423495 36854652

[CR15] Hamer M, Chida Y (2008) Active commuting and cardiovascular risk: a meta-analytic review. Prev Med 46(1):9–13. 10.1016/j.ypmed.2007.03.006 17475317

[CR16] Harryson L, Aléx L, Hammarström A (2016) I have surly passed a limit, it is simply too much’: women’s and men’s experiences of stress and wellbeing when living within a process of housework resignation. BMC Public Health 16:224. 10.1186/s12889-016-2920-5 PMC4779260 26944701

[CR17] Haukenes I, Hammarström A (2024) Workplace gender composition and sickness absence: a register-based study from Sweden. Scand J Public Health 52(6):678–684. 10.1177/14034948231176108 PMC11308254 37265198

[CR18] Holtermann A, Hansen JV, Burr H, Søgaard K, Sjøgaard G (2012) The health paradox of occupational and leisure-time physical activity. Br J Sports Med 46(4):291–295. 10.1136/bjsm.2010.079582 21459873

[CR19] Ji H, Gulati M, Huang TY, Kwan AC, Ouyang D, Ebinger JE, Casaletto K, Moreau KL, Skali H, Cheng S (2024) Sex differences in association of physical activity with all-cause and cardiovascular mortality. J Am Coll Cardiol 83(8):783–793. 10.1016/j.jacc.2023.12.019 PMC10984219 38383092

[CR20] Kalliolahti E, Gluschkoff K, Lanki T, Halonen JI, Salo P, Oksanen T, Ervasti J (2024) Associations between active commuting and sickness absence in finnish public sector cohort of 28 485 employees. Scand J Med Sci Sports 34(12):e70001. 10.1111/sms.70001 PMC11645541 39673325

[CR21] Kalliolahti E, Kurkela O, Salo P, Lanki T, Halonen JI, Oksanen T, Ervasti J (2025) Commute mode, commuting accidents and sickness absence among Finnish municipal employees. Eur J Pub Health 35(Supplement4):ckaf161254. 10.1093/eurpub/ckaf161.254

[CR22] Ketels M, Belligh T, De Bacquer D, Clays E (2023) The impact of leisure-time physical activity and occupational physical activity on sickness absence. A prospective study among people with physically demanding jobs. Scand J Work Environ Health 49(8):578–587. 10.5271/sjweh.4120 PMC10866622 37713180

[CR23] Kujala UM, Kaprio J, Sarna S, Koskenvuo M (1998) Relationship of leisure-time physical activity and mortality: the Finnish twin cohort. JAMA 279(6):440–444. 10.1001/jama.279.6.440 9466636

[CR25] Laaksonen M, Piha K, Martikainen P, Rahkonen O, Lahelma E (2009) Health-related behaviours and sickness absence from work. Occup Environ Med 66(12):840–847. 10.1136/oem.2008.039248 19934118

[CR24] Laaksonen M, Kaaria S-M, Leino-Arjas P, Lahelma E (2011) Different domains of health functioning as predictors of sickness absence—a prospective cohort study. Scand J Work Environ Health 37(3):213–218. http://www.jstor.org/stable/41151545 10.5271/sjweh.3131 21069253

[CR26] Lahti J, Lahelma E, Rahkonen O (2012) Changes in leisure-time physical activity and subsequent sickness absence: a prospective cohort study among middle-aged employees. Prev Med 55(6):618–622. 10.1016/j.ypmed.2012.10.006 23064133

[CR27] Lahti J, Reinikainen J, Kontto J, Zhou Z, Koskinen S, Laaksonen M, Partonen T, Elonheimo H, Lundqvist A, Tolonen H (2025) Work ability trends 2000–2020 and birth-cohort projections until 2040 in Finland. Scand J Public Health 53(1):62–70. 10.1177/14034948241228155 PMC11742703 38390654

[CR28] Lallukka T, Pietiläinen O, Jäppinen S, Laaksonen M, Lahti J, Rahkonen O (2020a) Factors associated with health survey response among young employees: a register-based study using online, mailed and telephone interview data collection methods. BMC Public Health 20(1):1–13. 10.1186/s12889-020-8241-8 PMC7003443 32024488

[CR29] Lallukka T, Shiri R, Pietiläinen O, Kausto J, Sumanen H, Halonen JI, Lahelma E, Rahkonen O, Mänty M, Kouvonen A (2020b) Timing of entry into paid employment, adverse physical work exposures and health: the young Helsinki health study. Int J Environ Res Public Health 17(21). 10.3390/ijerph17217854 PMC7662500 33120885

[CR30] Lehto MT, Kauppila T, Kautiainen H, Rahkonen O, Laine MK, Pitkälä K (2024) Who visits primary health care general practitioners and why? A register-based study in a Finnish city. Int J Circumpolar Health 83(1):2366034. 10.1080/22423982.2024.2366034 PMC11177702 38870400

[CR31] López-Bueno R, Sundstrup E, Vinstrup J, Casajús JA, Andersen LL (2020) High leisure-time physical activity reduces the risk of long-term sickness absence. Scand J Med Sci Sports 30(5):939–946. 10.1111/sms.13629 31986220

[CR32] Meh K, Sember V, Sorić M, Vähä-Ypyä H, Rocha P, Jurak G (2023) The dilemma of physical activity questionnaires: fitter people are less prone to over reporting. PLoS ONE 18(8):e0285357. 10.1371/journal.pone.0285357 PMC10468079 37647304

[CR33] Mytton OT, Panter J, Ogilvie D (2016) Longitudinal associations of active commuting with wellbeing and sickness absence. Prev Med 84:19–26. 10.1016/j.ypmed.2015.12.010 PMC4766368 26740344

[CR34] Niemelä MS, Kangas M, Ahola RJ, Auvinen JP, Leinonen A-M, Tammelin TH, Vaaramo ES, Keinänen-Kiukaanniemi SM, Korpelainen RI, Jämsä TJ (2019) Dose-response relation of self-reported and accelerometer-measured physical activity to perceived health in middle age-the Northern Finland birth cohort 1966 study. BMC Public Health 19(1):21. 10.1186/s12889-018-6359-8 PMC6322295 30612541

[CR35] Nyberg ST, Frank P, Pentti J, Alfredsson L, Ervasti J, Goldberg M, Knutsson A, Koskinen A, Lallukka T (2025) Health benefits of leisure-time physical activity by socioeconomic status, lifestyle risk, and mental health: a multicohort study. 124–135. 10.1016/S2468-2667(24)00300-1 PMC11803518 39909687

[CR36] Proper KI, van den Heuvel SG, De Vroome EM, Hildebrandt VH, Van der Beek AJ (2006) Dose-response relation between physical activity and sick leave. Br J Sports Med 40(2):173–178. 10.1136/bjsm.2005.022327 PMC2492021 16432007

[CR37] Punakallio A, Lusa S, Ala-Mursula L, Ek E, Nevanperä N, Remes J, Auvinen J, Seitsamo J, Karppinen J, Laitinen J (2019) Personal meaning of work and perceived work ability among middle-aged workers with physically strenuous work: a Northern Finland birth cohort 1966 study. Int Arch Occup Environ Health 92(3):371–381. 10.1007/s00420-019-01412-9 PMC6420453 30767053

[CR38] Quinlan C, Rattray B, Pryor D, Northey JM, Anstey KJ, Butterworth P, Cherbuin N (2021) The accuracy of self-reported physical activity questionnaires varies with sex and body mass index. PLoS ONE 16(8):e0256008. 10.1371/journal.pone.0256008 PMC8357091 34379676

[CR39] Saint-Maurice PF, Troiano RP, Berrigan D, Kraus WE, Matthews CE (2018) Volume of light versus moderate-to-vigorous physical activity: similar benefits for all-cause mortality? J Am Heart Association 7(7). 10.1161/JAHA.118.008815 PMC5907606 29610219

[CR40] Virtanen M, Ervasti J, Head J, Oksanen T, Salo P, Pentti J, Kouvonen A, Väänänen A, Suominen S, Koskenvuo M, Vahtera J, Elovainio M, Zins M, Goldberg M, Kivimäki M (2018) Lifestyle factors and risk of sickness absence from work: a multicohort study. Lancet Public Health 3(11):e545–e554. 10.1016/S2468-2667(18)30201-9 PMC6220357 30409406

