# Supplementary material for: Leisure-time and commuting physical activity and subsequent sickness absence among young and early midlife employees: a register-linked follow-up study 2017–2020
Source: Int Arch Occup Environ Health. 2026 Sep 1;99(7):43. doi: 10.1007/s00420-026-02234-2 (PMC13534223; doi:10.1007/s00420-026-02234-2)
Supplement: Supplementary file 1 [file 420_2026_2234_MOESM1_ESM.docx]

**Supplementary Table 1.** Number of total sickness absence (SA) days and short (≤7 days) or long (>7 days) SA spells per 10 person-years through 2017–2020 among the Helsinki Health Study. Leisure-time physical activity (LTPA) is categorized by volume and intensity of MET-hours per week in 2017 (n=3,618) and stratified by sex (women n=2,850); men n=768). Values are presented in means with standard deviations (SD) and medians with interquartile ranges (IQR).

| **LTPA** |  | **SA days** | **SA spells** | | **MET-hours** | | |
| --- | --- | --- | --- | --- | --- | --- | --- |
|  |  |  | Shorter | Longer |  | |  |
| **All participants** | N | Mean  (95% CI) | Mean  (95% CI) | Mean  (95% CI) | Range | Mean  (SD) | Median  (IQR) |
| High vigorous | 688 | 131.2 (118.6–143.9) | 26.7 (24.8–28.6) | 2.5 (2.1–2.8) | >70 | 88.9 (11.0) | 91.0 (21.9) |
| Vigorous | 943 | 119.7 (109.8–129.5) | 25.7 (24.1–27.3) | 2.1 (1.9–2.4) | 40–69.9 | 52.4 (8.9) | 51.5 (16.0) |
| Moderate-vigorous | 801 | 100.6 (91.6–109.6) | 24.8 (23.1–26.4) | 1.9 (1.6–2.2) | 14–39.9 | 26.6 (8.5) | 28.0 (13.0) |
| Moderate | 531 | 144.5 (128.7–160.3) | 29.9 (27.5–32.3) | 2.6 (2.2–3.0) | 14–39.9 | 24.0 (6.2) | 22.5 (8.0) |
| Inactive | 655 | 148.9 (134.2–163.4) | 30.5 (28.3–32.6) | 2.8 (2.4–3.2) | <14 | 8.0 (4.5) | 8.0 (8.0) |
|  |  | **SA days** | **SA spells** | | **MET-hours** | | |
|  |  |  | Shorter | Longer |  |  |  |
| **Women** | N | Mean  (95% CI) | Mean  (95% CI) | Mean  (95% CI) | Range | Mean  (SD) | Median  (IQR) |
| High vigorous | 506 | 141.3 (127.3–155.2) | 28.5 (26.4–30.6) | 2.6 (2.3–3.0) | >70 | 88.5 (10.9) | 90.0 (21.9) |
| Vigorous | 734 | 127.1 (116.4–137.9) | 27.2 (25.5–28.9) | 2.3 (2.0–2.6) | 40–69.9 | 51.2 (8.9) | 51.0 (15.5) |
| Moderate-vigorous | 636 | 107.9 (98.0–117.9) | 26.1 (24.4–27.9) | 2.0 (1.7–2.3) | 14–39.9 | 26.8 (8.2) | 28.0 (13.5) |
| Moderate | 443 | 151.4 (134.8–168.1) | 31.1 (28.6–33.6) | 2.7 (2.3–3.2) | 14–39.9 | 23.9 (6.2) | 22.0 (9.0) |
| Inactive | 531 | 159.3 (143.3–175.3) | 32.1 (29.8–34.4) | 3.0 (2.6–3.4) | <14 | 8.3 (4.3) | 9.0 (7.0) |
|  |  | **SA days** | **SA spells** | | **MET-hours** | | |
|  |  |  | Shorter | Longer |  |  |  |
| **Men** | N | Mean  (95% CI) | Mean  (95% CI) | Mean  (95% CI) | Range | Mean  (SD) | Median  (IQR) |
| High vigorous | 182 | 100.1 (87.8–112.4) | 21.6 (19.6–23.6) | 1.9 (1.6–2.3) | >70 | 89.9 (11.3) | 95.0 (21.9) |
| Vigorous | 209 | 90.1 (79.6–100.5) | 20.6 (18.8–22.3) | 1.7 (1.4–2.0) | 40–69.9 | 53.6 (8.9) | 53.0 (16.3) |
| Moderate-vigorous | 165 | 76.5 (67.4–85.5) | 19.8 (18.0–21.6) | 1.5 (1.2–1.7) | 14–39.9 | 25.6 (9.5) | 26.0 (14.0) |
| Moderate | 88 | 107.3 (92.4–122.2) | 23.5 (21.1–26.0) | 2.0 (1.6–2.4) | 14–39.9 | 24.8 (6.3) | 24.0 (10.0) |
| Inactive | 124 | 112.9 (98.6–127.1) | 24.3 (22.0–26.6) | 2.2 (1.8–2.6) | <14 | 6.8 (4.9) | 7.0 (9.7) |

**Supplementary Table 2.** Number of total sickness absence (SA) days and short (≤7 days) or long (>7 days) SA spells per 10 person-years through 2017–2020 among the Helsinki Health Study. Active commuting (AC) is categorized by volume and intensity of MET-hours per week in 2017 (n=3,513) and stratified by sex (women n=2,773); men n=740). Values are presented in means with standard deviations (SD) and medians with interquartile ranges (IQR).

| **AC** |  | **SA days** | **SA spells** | | **MET-hours** | | |
| --- | --- | --- | --- | --- | --- | --- | --- |
|  |  |  | Shorter | Longer |  | |  |
| **All participants** | N | Mean  (95% CI) | Mean  (95% CI) | Mean  (SD) | Range | Mean  (SD) | Median  (IQR) |
| Vigorous | 882 | 125.5 (114.7–136.2) | 26.8 (25.1–28.5) | 2.2 (1.9–2.5) | ≥20 | 35.2 (14.6) | 30.0 (16.0) |
| Moderate-vigorous | 890 | 122.6 (112.3–133.0) | 26.6 (24.9–28.2) | 2.3 (2.0–2.6) | 9–19.9 | 13.2 (3.0) | 12.6 (5.3) |
| Low moderate | 827 | 110.8 (101.0–120.5) | 27.3 (25.6–29.1) | 2.2 (1.9–2.4) | 3–8.9 | 6.1 (1.8) | 6.0 (4.0) |
| Inactive | 914 | 145.8 (133.6–158.1) | 27.7 (26.0–29.4) | 2.6 (2.3–2.9) | <3 | 0.9 (1.1) | 1.0 (2.0) |
|  |  | **SA days** | **SA spells** | | **MET-hours** | | |
|  |  |  | Shorter | Longer |  |  |  |
| **Women** | N | Mean  (95% CI) | Mean  (95% CI) | Mean  (95% CI) | Range | Mean  (SD) | Median  (IQR) |
| Vigorous | 686 | 133.3 (121.7–145.0) | 28.2 (26.4–30.0) | 2.4 (2.1–2.7) | ≥20 | 34.7 (14.2) | 30.0 (16.0) |
| Moderate-vigorous | 721 | 130.8 (119.4–142.2) | 28.0 (26.2–29.7) | 2.4 (2.1–2.7) | 9–19.9 | 13.1 (3.0) | 12.0 (5.3) |
| Low moderate | 666 | 117.8 (107.2–128.4) | 28.7 (26.8–30.6) | 2.3 (2.0–2.6) | 3–8.9 | 6.1 (1.8) | 6.0 (4.0) |
| Inactive | 700 | 156.4 (142.8–170.0) | 29.4 (27.6–31.3) | 2.8 (2.4–3.1) | <3 | 1.0 (1.1) | 1.0 (2.0) |
|  |  | **SA days** | **SA spells** | | **MET-hours** | | |
|  |  |  | Shorter | Longer |  |  |  |
| **Men** | N | Mean  (95% CI) | Mean  (95% CI) | Mean  (95% CI) | Range | Mean  (SD) | Median  (IQR) |
| Vigorous | 196 | 94.2 (83.0–105.4) | 21.4 (19.5–23.2) | 1.7 (1.4–2.0) | ≥20 | 36.8 (16.0) | 30.3 (19.8) |
| Moderate-vigorous | 169 | 92.4 (81.5–103.3) | 21.2 (19.3–23.0) | 1.8 (1.5–2.1) | 9–19.9 | 13.5 (3.1) | 13.0 (4.5) |
| Low moderate | 161 | 83.2 (73.2–93.3) | 21.7 (19.8–23.7) | 1.7 (1.4–2.0) | 3–8.9 | 5.9 (1.8) | 6.0 (4.0) |
| Inactive | 214 | 110.5 (97.7–123.3) | 22.3 (20.4–24.2) | 2.0 (1.7–2.4) | <3 | 0.7 (0.9) | 0 (1.0) |

**Supplementary Table 3.** Incidence rate ratios (IRRs) of total sickness absence (SA) days and short (≤7 days) or long (>7 days) SA spells through 2017–2020 by leisure-time physical activity (LTPA) groups among women (N=2,850) (ref. inactive). Estimates are derived from negative binomial regression models..

| **Women (n=2,850)** |  |  |  |  |  |  |
| --- | --- | --- | --- | --- | --- | --- |
| LTPA | Age | *Occupational class | *Mental strenuousness of work | *Physical strenuousness of work | *BMI | *Smoking |
| **SA days** |  |  |  |  |  |  |
| High vigorous | 0.94 (0.81–1.10) | 0.98 (0.84–1.14) | 0.91 (0.78–1.06) | 0.94 (0.80–1.09) | 1.02 (0.87–1.19) | 0.97 (0.83–1.13) |
| Vigorous | 0.85 (0.73–0.97) | 0.92 (0.79–1.05) | 0.82 (0.71–0.94) | 0.85 (0.74–0.98) | 0.95 (0.82–1.09) | 0.85 (0.74–0.98) |
| MVPA | 0.67 (0.58–0.78) | 0.72 (0.63–0.84) | 0.68 (0.59–0.79) | 0.68 (0.59–0.79) | 0.75 (0.65–0.87) | 0.69 (0.60–0.80) |
| Moderate | 0.99 (0.85–1.17) | 1.01 (0.87–1.19) | 1.00 (0.86–1.17) | 0.98 (0.84–1.15) | 1.02 (0.87–1.19) | 0.99 (0.85–1.16) |
| Inactive | 1 | 1 | 1 | 1 | 1 | 1 |
| **SA short spells** |  |  |  |  |  |  |
| High vigorous | 0.91 (0.81–1.01) | 0.96 (0.86–1.07) | 0.92 (0.83–1.03) | 0.92 (0.83–1.03) | 0.99 (0.89–1.11) | 0.94 (0.84–1.05) |
| Vigorous | 0.86 (0.78–0.95) | 0.94 (0.85–1.03) | 0.87 (0.79–0.97) | 0.88 (0.79–0.97) | 0.95 (0.86–1.05) | 0.89 (0.80–0.98) |
| MVPA | 0.81 (0.73–0.90) | 0.89 (0.80–0.99) | 0.82 (0.74–0.91) | 0.83 (0.75–0.92) | 0.88 (0.79–0.98) | 0.84 (0.75–0.93) |
| Moderate | 0.99 (0.89–1.11) | 1.02 (0.92–1.14) | 1.01 (0.90–1.14) | 1.01 (0.90–1.13) | 1.01(0.90–1.13) | 1.00 (0.89–1.12) |
| Inactive | 1 | 1 | 1 | 1 | 1 | 1 |
| **SA long spells** |  |  |  |  |  |  |
| High vigorous | 0.89 (0.72–1.10) | 0.93 (0.75–1.16) | 0.90 (0.72–1.11) | 0.89 (0.72–1.10) | 0.99 (0.80–1.24) | 0.91 (0.73–1.13) |
| Vigorous | 0.79 (0.64–0.96) | 0.85 (0.70–1.04) | 0.79 (0.65–0.97) | 0.80 (0.65–0.97) | 0.88 (0.72–1.08) | 0.81 (0.66–0.98) |
| MVPA | 0.63 (0.39–0.99) | 0.76 (0.61–0.94) | 0.69 (0.56–0.86) | 0.70 (0.57–0.87) | 0.76 (0.61–0.94) | 0.70 (0.57–0.87) |
| Moderate | 0.98 (0.58–1.64) | 0.93 (0.75–1.16) | 0.91 (0.73–1.14) | 0.91 (0.73–1.13) | 0.92 (0.74–1.15) | 0.91 (0.73–1.14) |
| Inactive | 1 | 1 | 1 | 1 | 1 | 1 |

* Age adjusted.

Incidence rate ratios (IRRs) and 95% CI adjusted for age, occupational class and mental and physical strenuousness of work, body mass index (BMI) and smoking.

MVPA; moderate-vigorous physical activity

**Supplementary Table 4.** Incidence rate ratios (IRRs) of total sickness absence (SA) days and short (≤7 days) or long (>7 days) SA spells through 2017–2020 by leisure-time physical activity (LTPA) groups among men (N=768) (ref. inactive). Estimates are derived from negative binomial regression models.

| **Men (n=768)** |  |  |  |  |  |  |
| --- | --- | --- | --- | --- | --- | --- |
| LTPA | Age | *Occupational class | *Mental strenuousness of work | *Physical strenuousness of work | *BMI | *Smoking |
| **SA days** |  |  |  |  |  |  |
| High vigorous | 0.68 (0.50–0.93) | 0.76 (0.56–1.04) | 0.65 (0.47–0.89) | 0.69 (0.51–0.95) | 0.78 (0.57–1.08) | 0.72 (0.53–0.99) |
| Vigorous | 0.60 (0.44–0.81) | 0.68 (0.50–0.92) | 0.58 (0.43–0.78) | 0.62 (0.46–0.84) | 0.70 (0.51–0.95) | 0.63 (0.47–0.86) |
| MVPA | 0.65 (0.47–0.89) | 0.77 (0.56–1.05) | 0.62 (0.45–0.85) | 0.69 (0.50–0.95) | 0.72 (0.53–0.99) | 0.67 (0.49–0.92) |
| Moderate | 0.77 (0.53–1.12) | 0.91 (0.62–1.32) | 0.74 (0.50–1.08) | 0.81 (0.55–1.18) | 0.82 (0.56–1.19) | 0.80 (0.55–1.16) |
| Inactive | 1 | 1 | 1 | 1 | 1 | 1 |
| **SA short spells** |  |  |  |  |  |  |
| High vigorous | 0.74 (0.59–0.94) | 0.80 (0.64–1.01) | 0.75 (0.60–0.95) | 0.76 (0.61–0.96) | 0.78 (0.61–0.98) | 0.94 (0.84–1.05) |
| Vigorous | 0.77 (0.61–0.98) | 0.77 (0.62–0.96) | 0.73 (0.58–0.91) | 0.74 (0.59–0.92) | 0.74 (0.59–0.93) | 0.89 (0.80–0.98) |
| MVPA | 0.77 (0.61–0.98) | 0.83 (0.65–1.05) | 0.77 (0.60–0.97) | 0.78 (0.62–0.99) | 0.79 (0.62–1.00) | 0.84 (0.75–0.93) |
| Moderate | 0.82 (0.62–1.08) | 0.84 (0.64–1.10) | 0.82 (0.62–1.09) | 0.83 (0.63–1.10) | 0.83(0.63–1.10) | 1.00 (0.89–1.12) |
| Inactive | 1 | 1 | 1 | 1 | 1 | 1 |
| **SA long spells** |  |  |  |  |  |  |
| High vigorous | 0.84 (0.54–1.30) | 0.90 (0.58–1.38) | 0.78 (0.51–1.22) | 0.84 (0.54–1.29) | 0.90 (0.58–1.40) | 0.84 (0.54–1.31) |
| Vigorous | 0.65 (0.42–0.99) | 0.71 (0.46–1.10) | 0.61 (0.40–0.95) | 0.68 (0.44–1.05) | 0.70 (0.45–1.09) | 0.65 (0.42–1.01) |
| MVPA | 0.63 (0.39–0.99) | 0.73 (0.46–1.15) | 0.61 (0.38–0.97) | 0.67 (0.42–1.07) | 0.68 (0.43–1.08) | 0.63 (0.40–1.01) |
| Moderate | 0.98 (0.58–1.64) | 1.10 (0.65–1.84) | 0.94 (0.55–1.58) | 1.02 (0.61–1.70) | 0.99 (0.59–1.67) | 0.98 (0.58–1.65) |
| Inactive | 1 | 1 | 1 | 1 | 1 | 1 |

* Age adjusted.

Incidence rate ratios (IRRs) and 95% CI adjusted for age, occupational class and mental and physical strenuousness of work, body mass index (BMI) and smoking.

MVPA; moderate-vigorous physical activity

**Supplementary Table 5.** Incidence rate ratios (IRRs) of total sickness absence (SA) days and short (≤7 days) or long (>7 days) SA spells through 2017–2020 by active commuting (AC) groups among women (N=2,773) (ref. inactive). Estimates are derived from negative binomial regression models.

| AC | Age | *Occupational class | *Mental strenuousness of work | *Physical strenuousness of work | *BMI | *Smoking |
| --- | --- | --- | --- | --- | --- | --- |
| **SA days** |  |  |  |  |  |  |
| Vigorous | 0.87 (0.76–0.99) | 0.90 (0.78–1.02) | 0.86 (0.75–0.98) | 0.88 (0.77–1.00) | 0.91 (0.80–1.04) | 0.86 (0.75–0.98) |
| MVPA | 0.80 (0.70–0.91) | 0.81 (0.71–0.92) | 0.82 (0.72–0.93) | 0.82 (0.72–0.94) | 0.84 (0.74–0.95) | 0.80 (0.70–0.91) |
| Low moderate | 0.73 (0.63–0.83) | 0.74 (0.65–0.85) | 0.75 (0.66–0.86) | 0.74 (0.65–0.85) | 0.74 (0.65–0.85) | 0.73 (0.64–0.83) |
| Inactive | 1 | 1 | 1 | 1 | 1 | 1 |
| **SA short spells** |  |  |  |  |  |  |
| Vigorous | 0.98 (0.90–1.08) | 0.99 (0.91–1.09) | 0.98 (0.90–1.08) | 0.98 (0.90–1.08) | 1.02 (0.93–1.13) | 0.99 (0.90–1.09) |
| MVPA | 0.93 (0.85–1.02) | 0.96 (0.88–1.06) | 0.93 (0.85–1.03) | 0.95 (0.86–1.04) | 0.96 (0.88–1.06) | 0.94 (0.85–1.03) |
| Low moderate | 0.97 (0.88–1.06) | 0.98 (0.89–1.08) | 0.97 (0.88–1.07) | 0.97 (0.88–1.07) | 0.98 (0.89–1.08) | 0.97 (0.90–1.09) |
| Inactive | 1 | 1 | 1 | 1 | 1 | 1 |
| **SA long spells** |  |  |  |  |  |  |
| Vigorous | 0.85 (0.70–1.04) | 0.87 (0.72–1.06) | 0.87 (0.71–1.05) | 0.86 (0.71–1.05) | 0.90 (0.74–1.10) | 0.85 (0.71–1.04) |
| MVPA | 0.84 (0.70–1.02) | 0.88 (0.72–1.06) | 0.86 (0.71–1.04) | 0.87 (0.72–1.06) | 0.88 (0.73–1.07) | 0.85 (0.71–1.03) |
| Low moderate | 0.82 (0.67–0.99) | 0.84 (0.69–1.16) | 0.84 (0.69–1.02) | 0.84 (0.69–1.02) | 0.83 (0.68–1.01) | 0.83 (0.68–1.00) |
| Inactive | 1 | 1 | 1 | 1 | 1 | 1 |

* Age adjusted.

Incidence rate ratios (IRRs) and 95% CI adjusted for age, occupational class and mental and physical strenuousness of work, body mass index (BMI) and smoking.

MVPA; moderate-vigorous physical activity

**Supplementary Table 6.** Incidence rate ratios (IRRs) of total sickness absence (SA) days and short (≤7 days) or long (>7 days) SA spells through 2017–2020 by active commuting (AC) groups among men (N=740) (ref. inactive). Estimates are derived from negative binomial regression models adjusted for follow-up time (offset).

| AC | Age | *Occupational class | *Mental strenuousness of work | *Physical strenuousness of work | *BMI | *Smoking |
| --- | --- | --- | --- | --- | --- | --- |
| **SA days** |  |  |  |  |  |  |
| Vigorous | 0.78 (0.60–1.03) | 0.86 (0.66–1.12) | 0.77 (0.58–1.00) | 0.79 (0.61–1.18) | 0.91 (0.69–1.19) | 0.81 (0.62–1.06) |
| MVPA | 0.95 (0.72–1.27) | 1.07 (0.81–1.41) | 0.95 (0.71–1.26) | 1.03 (0.78–1.37) | 1.06 (0.80–1.41) | 0.99 (0.75–1.31) |
| Low moderate | 0.83 (0.62–1.10) | 0.96 (0.72–1.27) | 0.82 (0.61–1.09) | 0.88 (0.66–1.18) | 0.97 (0.73–1.30) | 0.81 (0.62–1.06) |
| Inactive | 1 | 1 | 1 | 1 | 1 | 1 |
| **SA short spells** |  |  |  |  |  |  |
| Vigorous | 0.84 (0.65–1.02) | 0.88 (0.72–1.07) | 0.83 (0.68–1.02) | 0.84 (0.69–1.03) | 0.86 (0.70–1.05) | 0.86 (0.70–1.05) |
| MVPA | 0.98 (0.80–1.21) | 1.02 (0.83–1.24) | 0.98 (0.79–1.20) | 1.03 (0.84–1.27) | 1.02 (0.82–1.25) | 1.00 (0.82–1.23) |
| Low moderate | 0.94 (0.76–1.17) | 0.99 (0.81–1.22) | 0.95 (0.77–1.17) | 0.97 (0.78–1.20) | 0.97 (0.79–1.21) | 0.94 (0.76–1.16) |
| Inactive | 1 | 1 | 1 | 1 | 1 | 1 |
| **SA long spells** |  |  |  |  |  |  |
| Vigorous | 0.86 (0.58–1.28) | 0.90 (0.61–1.33) | 0.84 (0.56–1.24) | 0.89 (0.60–1.31) | 0.96 (0.65–1.42) | 0.88 (0.59–1.30) |
| MVPA | 1.05 (0.71–1.56) | 1.18 (0.80–1.75) | 1.03 (0.69–1.54) | 1.20 (0.80–1.78) | 1.17 (0.79–1.75) | 1.08 (0.72–1.60) |
| Low moderate | 0.83 (0.54–1.27) | 0.91 (0.59–1.39) | 0.84 (0.55–1.28) | 0.92 (0.60–1.40) | 0.95 (0.61–1.46) | 0.83 (0.54–1.27) |
| Inactive | 1 | 1 | 1 | 1 | 1 | 1 |

* Age adjusted.

Rate ratios (RRs) and 95% CI adjusted for age, occupational class and mental and physical strenuousness of work, body mass index (BMI) and smoking. MVPA; moderate-vigorous physical activity

**Supplementary Table 7.** Predicted sickness absence (SA) days, short and long SA spells per 10 person-years by leisure-time physical activity (LTPA) and sex. Values are marginal means with 95% CIs and standard errors (SE). Estimates are derived from negative binomial regression models including sex and LTPA interactions, adjusted for age and follow-up time at work (offset).

| LTPA  Sex | SA days  LTPA category | Predicted mean, 95% CI | SE |
| --- | --- | --- | --- |
| Women (n=2,850) | High vigorous | 145.6 (129.3–161.8) | 8.27 |
| Women | Vigorous | 130.4 (118.3–142.5) | 6.17 |
| Women | Moderate-vigorous | 104.0 (93.7–114.3) | 5.27 |
| Women | Moderate | 153.4 (135.2–171.6) | 9.30 |
| Women | Inactive | 153.8 (137.1–170.5) | 8.52 |
| Men (n=768) | High vigorous | 90.8 (73.9–107.7) | 8.61 |
| Men | Vigorous | 80.5 (66.6–94.4) | 7.11 |
| Men | Moderate-vigorous | 87.2 (70.2–104.2) | 8.68 |
| Men | Moderate | 102.6 (74.9–130.3) | 14.11 |
| Men | Inactive | 134.6 (104.6–164.7) | 15.35 |
|  |  |  |  |
| LTPA | Short SA spells |  |  |
|  |  |  |  |
| Sex | LTPA category | Predicted mean, 95% CI | SE |
| Women (n=2,850) | High vigorous | 28.8 (26.5–31.2) | 1.19 |
| Women | Vigorous | 27.3 (25.5–29.2) | 0.94 |
| Women | Moderate-vigorous | 25.9 (24.0–27.7) | 0.96 |
| Women | Moderate | 31.6 (28.9–34.4) | 1.39 |
| Women | Inactive | 31.8 (29.3–34.3) | 1.27 |
| Men (n=768) | High vigorous | 20.3 (17.5–23.2) | 1.45 |
| Men | Vigorous | 19.9 (17.3–22.4) | 1.32 |
| Men | Moderate-vigorous | 20.9 (17.8–24.0) | 1.56 |
| Men | Moderate | 22.2 (17.7–26.7) | 2.28 |
| Men | Inactive | 27.1 (22.6–31.5) | 2.26 |
|  |  |  |  |
| LTPA | Long SA spells |  |  |
|  |  |  |  |
| Sex | LTPA category | Predicted mean, 95% CI | SE |
| Women (n=2,850) | High vigorous | 2.6 (2.2–3.1) | 0.21 |
| Women | Vigorous | 2.3 (2.1–2.6) | 0.16 |
| Women | Moderate-vigorous | 2.0 (1.7–2.3) | 0.16 |
| Women | Moderate | 2.7 (2.2–3.1) | 0.23 |
| Women | Inactive | 3.0 (2.5–3.4) | 0.22 |
| Men (n=768) | High vigorous | 1.9 (1.4–2.5) | 0.28 |
| Men | Vigorous | 1.5 (1.1–1.9) | 0.22 |
| Men | Moderate-vigorous | 1.4 (1.0–1.9) | 0.24 |
| Men | Moderate | 2.3 (1.3–3.2) | 0.46 |
| Men | Inactive | 2.3 (1.6–3.1) | 0.39 |

**Supplementary Table 8.** Predicted sickness absence (SA) days, short and long SA spells by active commuting (AC) and sex. Values are marginal means with 95% CIs and standard errors (SE). Estimates are derived from negative binomial regression models including gender and AC interactions, adjusted for age and follow-up time at work (offset).

| AC  Sex | SA days  AC category | Predicted mean, 95% CI | SE |
| --- | --- | --- | --- |
| Women (n=2,773) | Vigorous | 138.0 (124.7–151.3) | 6.78 |
| Women | Moderate-vigorous | 126.7 (114.8–138.5) | 6.04 |
| Women | Low moderate | 115.5 (104.3–126.7) | 5.72 |
| Women | Inactive | 159.0 (143.9–174.2) | 7.73 |
| Men (n=740) | Vigorous | 85.0 (69.7–100.3) | 7.80 |
| Men | Moderate-vigorous | 101.5 (82.0–121.1) | 9.98 |
| Men | Low moderate | 88.1 (70.4–105.8) | 9.03 |
| Men | Inactive | 108.3 (89.7–126.9) | 9.48 |
|  |  |  |  |
| AC | Short SA spells |  |  |
|  |  |  |  |
| Sex | AC category | Predicted mean, 95% CI | SE |
| Women (n=2,773) | Vigorous | 29.0 (27.0–31.1) | 1.03 |
| Women | Moderate-vigorous | 27.5 (25.6–29.3) | 0.95 |
| Women | Low Moderate | 28.5 (26.5–30.5) | 1.02 |
| Women | Inactive | 29.5 (27.5–31.6) | 1.04 |
| Men (n=740) | Vigorous | 19.3 (16.7–21.9) | 1.33 |
| Men | Moderate-vigorous | 22.7 (19.5–25.9) | 1.64 |
| Men | Low moderate | 21.9 (18.6–25.1) | 1.65 |
| Men | Inactive | 23.0 (20.1–25.9) | 1.50 |
|  |  |  |  |
| AC | Long SA spells |  |  |
|  |  |  |  |
| Sex | AC category | Predicted mean, 95% CI | SE |
| Women (n=2,773) | Vigorous | 2.4 (2.1–2.7) | 0.17 |
| Women | Moderate-vigorous | 2.4 (2.0–2.7) | 0.17 |
| Women | Low moderate | 2.3 (2.0–2.6) | 0.17 |
| Women | Inactive | 2.8 (2.4–3.2) | 0.19 |
| Men (n=740) | Vigorous | 1.7 (1.2–2.2) | 0.25 |
| Men | Moderate-vigorous | 2.0 (1.4–2.6) | 0.30 |
| Men | Low moderate | 1.6 (1.1–2.1) | 0.27 |
| Men | Inactive | 2.0 (1.4–2.5) | 0.26 |

**Supplementary File 1.** Questionnaire items used to assess leisure-time physical activity and active commuting in 2017 among the Helsinki Health Study.

**Supplementary File 2.** STROBE Statement—Checklist of items that should be included in reports of *cohort studies*

**Päivärinne et al. “Leisure-time and commuting physical activity and subsequent sickness absence among young and early midlife employees: a register-linked follow-up study 2017–2020”**

|  | Item No | Recommendation |
| --- | --- | --- |
| Title and abstract | 1 | (*a*) Indicate the study’s design with a commonly used term in the title or the abstract **(Title; page 2, rows 90–91)** |
|  |  | (*b*) Provide in the abstract an informative and balanced summary of what was done and what was found |
| Introduction | | |
| Background/rationale | 2 | Explain the scientific background and rationale for the investigation being reported **(Pages 1–2, rows 70–80)** |
| Objectives | 3 | State specific objectives, including any prespecified hypotheses **(pages 1–2, rows 31–80)** |
| Methods | | |
| Study design | 4 | Present key elements of study design early in the paper **(pages 2–4, rows 86–172)** |
| Setting | 5 | Describe the setting, locations, and relevant dates, including periods of recruitment, exposure, follow-up, and data collection **(page 2, rows 86–115)** |
| Participants | 6 | (*a*) Give the eligibility criteria, and the sources and methods of selection of participants. Describe methods of follow-up (**pages 2 & 4, Figure 1)** |
|  |  | (*b*) For matched studies, give matching criteria and number of exposed and unexposed |
| Variables | 7 | Clearly define all outcomes, exposures, predictors, potential confounders, and effect modifiers. Give diagnostic criteria, if applicable **(pages 3–4, rows 119–172)** |
| Data sources/ measurement | 8* | For each variable of interest, give sources of data and details of methods of assessment (measurement). Describe comparability of assessment methods if there is more than one group. |
| Bias | 9 | Describe any efforts to address potential sources of bias **(pages 11–12, rows 375–395)** |
| Study size | 10 | Explain how the study size was arrived at **(Figure 1)** |
| Quantitative variables | 11 | Explain how quantitative variables were handled in the analyses. If applicable, describe which groupings were chosen and why **(Page 3, rows 119–154; page 4, rows 190–193)** |
| Statistical methods | 12 | (*a*) Describe all statistical methods, including those used to control for confounding **(Page 4, rows 174–204)** |
|  |  | (*b*) Describe any methods used to examine subgroups and interactions (P**age 4, rows 189–193)** |
|  |  | (*c*) Explain how missing data were addressed **(Page 2, rows 108–110)** |
|  |  | (*d*) If applicable, explain how loss to follow-up was addressed **(Page 3, rows 144–147)** |
|  |  | (*e*) Describe any sensitivity analyses **(Supplementary tables 3–6)** |
| Results | | |
| Participants | 13* | (a) Report numbers of individuals at each stage of study—eg numbers potentially eligible, examined for eligibility, confirmed eligible, included in the study, completing follow-up, and analysed **(Figure 1)** |
|  |  | (b) Give reasons for non-participation at each stage **(Figure 1)** |
|  |  | (c) Consider use of a flow diagram **(Figure 1)** |
| Descriptive data | 14* | (a) Give characteristics of study participants (eg demographic, clinical, social) and information on exposures and potential confounders **(pages 2–4, rows 87–171)** |
|  |  | (b) Indicate number of participants with missing data for each variable of interest **(Table 1)** |
|  |  | (c) Summarise follow-up time (eg, average and total amount) **(Page 3, rows 144–147)** |
| Outcome data | 15* | Report numbers of outcome events or summary measures over time **(Table 2; Figures 2 & 3, Supplementary tables 3–8)** |
| Main results | 16 | (*a*) Give unadjusted estimates and, if applicable, confounder-adjusted estimates and their precision (eg, 95% confidence interval). Make clear which confounders were adjusted for and why they were included **(Pages 1–2, rows 71–81; Supplementary tables 3–8)** |
|  |  | (*b*) Report category boundaries when continuous variables were categorized **(Page 3, rows 131–138)** |
|  |  | (*c*) If relevant, consider translating estimates of relative risk into absolute risk for a meaningful time period |
| Other analyses | 17 | Report other analyses done—eg analyses of subgroups and interactions, and sensitivity analyses **(Supplementary tables 5–6)** |
| Discussion | | |
| Key results | 18 | Summarise key results with reference to study objectives **(Page 10, rows 291–299)** |
| Limitations | 19 | Discuss limitations of the study, taking into account sources of potential bias or imprecision. Discuss both direction and magnitude of any potential bias **(Pages 11–12, rows 375–395)** |
| Interpretation | 20 | Give a cautious overall interpretation of results considering objectives, limitations, multiplicity of analyses, results from similar studies, and other relevant evidence **(Page 12, rows 411–422)** |
| Generalisability | 21 | Discuss the generalisability (external validity) of the study results **(Pages 11–12, rows 375–422)** |
| Other information | | |
| Funding | 22 | Give the source of funding and the role of the funders for the present study and, if applicable, for the original study on which the present article is based **(Title page)** |

*Give information separately for exposed and unexposed groups.

**Note:** An Explanation and Elaboration article discusses each checklist item and gives methodological background and published examples of transparent reporting. The STROBE checklist is best used in conjunction with this article (freely available on the Web sites of PLoS Medicine at http://www.plosmedicine.org/, Annals of Internal Medicine at http://www.annals.org/, and Epidemiology at http://www.epidem.com/). Information on the STROBE Initiative is available at http://www.strobe-statement.org.
